# On-Chip Photonic Synapses with All-Optical Memory and Neural Network Computation

**DOI:** 10.3390/mi14010074

**Published:** 2022-12-27

**Authors:** Lulu Zhang, Yongzhi Zhang, Furong Liu, Qingyuan Chen, Yangbo Lian, Quanlong Ma

**Affiliations:** 1 MOE Key Laboratory of Trans-Scale Laser Manufacturing Technology, Beijing University of Technology, Beijing 100124, China; 2 Beijing Engineering Research Center of Laser Technology, Beijing University of Technology, Beijing 100124, China; 3 Institute of Laser Engineering, Faculty of Materials and Manufacturing, Beijing University of Technology, Beijing 100124, China

**Keywords:** phase-change materials, photonic neural network, photonic synapse, optical memory, digital recognition

## Abstract

Inspired by the human brain, neural network computing was expected to break the bottleneck of traditional computing, but the integrated design still faces great challenges. Here, a readily integrated membrane-system photonic synapse was demonstrated. By pre-pulse training at 1064 nm (cutoff wavelength), the photonic synapse can be regulated both excitatory and inhibitory at tunable wavelengths (1200–2000 nm). Furthermore, more weights and memory functions were shown through the photonic synapse integrated network. Additionally, the digital recognition function of the single-layer perceptron neural network constructed by photonic synapses has been successfully demonstrated. Most of the biological synaptic functions were realized by the photonic synaptic network, and it had the advantages of compact structure, scalable, adjustable wavelength, and so on, which opens up a new idea for the study of the neural synaptic network.

## 1. Introduction

**Figure 1:**
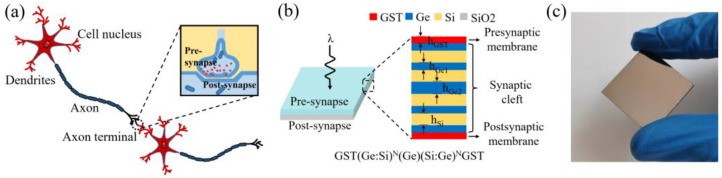
(**a**) The schematic diagram of biological synapses. (**b**) The schematic diagram of on-chip photonic synaptic structures. (**c**) The physical picture of on-chip photonic synaptic.

Over the previous decades, Von Neumann computing systems [1,2], which consist of independent memory units (memory) and computing units (CPU), are facing the challenge of information capacity. In contrast, the human brain is composed of a network of neural synapses, as shown in Figure 1a, each of which can be used as a memory and computation unit capable of processing a large amount of information [3,4]. Inspired by the human brain, the neural synaptic computing network that integrates memory and computing has been widely concerned [5]. Recently, several electronic devices that achieve synaptic function have been reported, such as electrically-induced metal oxides [6], transistors [7] and memristors [8,9], among others. However, electronic synapses are triggered by electrical signals, and the corresponding neural synaptic network is usually limited by Moore’s law [10]. Compared with electronic synapses, photonic synapses may be a more favorable choice for increasing computational speed [11,12]. Gholipour et al. [13] proposed photonic synaptic devices based on microfibers for the first time in 2015, and its high-speed signal transmission, similar to that of the human brain, was demonstrated.

With further research, more and more new materials and structures have been discovered, such as inorganic perovskite [14], black phosphorus [15] and phase-change materials [16], etc. Among them, phase-change materials (PCMs) are one of the ideal candidates in the field of integrated photonics and have become a hot topic in the research of optical switches [17], optical modulators [18], optical computing [19,20] and optical neural networks [21]. Cheng et al. [22] demonstrated a photonic synapse based on an integrated PCMs-Si waveguide in 2017, where the weight of the synapse can be set by the light pulse. Although some basic functions of information transfer and weight regulation have been implemented by the above-mentioned photonic synaptic devices, the structure of the neural synaptic network has not been constructed. In 2019, Feldmann et al. [23] demonstrated the synaptic network based on a PCMs-ring resonator and demonstrated its pattern recognition function. Further, more and more photonic synaptic neural networks based on PCMs had been studied. In 2020, Miscuglio et al. [24] proposed a photonic memory based on mixed PCMs-silicon Mach Zender to simulate multi-layer perceptron neural networks. Wu et al. [25] demonstrated the implementation of a neural network based on waveguide-PCMs metasurfaces in 2021. In 2021, Zhang et al. [26] proposed a photonic synapse based on a PCMs-slot ridge waveguide and constructed a neural network to realize digit recognition. Although photonic synaptic neural networks have made some important progress, the realization of photonic synapses depends on the hybrid waveguide-PCMs structure. The structure has the advantages of small size and high precision, but it has the disadvantages of fixed transmission wavelength, low coupling efficiency and difficult integration. The future development of photonic synapses should be in the direction of easy integration, multi-band and multi-weight.

In this work, a membrane-system structure of on-chip photonic synapses was proposed, consisting of Ge_2_Sb_2_Te_5_ (GST) films embedded at both ends of Ge/Si films. The structure can be trained at 1064 nm (cutoff wavelength) and exhibited both excitatory and inhibitory regulations at tunable wavelengths (1200–2000 nm). Then, more weights were shown through the integration of photonic synapses, and the memory function of the neurons was demonstrated. In addition, the constructed single-layer perceptron neural network realized the function of digital recognition. The photonic synapse based on this structure had the advantages of compact, scalable and tunable wavelength, and can be combined with the existing mature waveguide or fiber structure, so it had wide applicability in neural synapse computation.

## 2. Structures and Methods

### 2.1. Structure of On-Chip Photonic Synapses

Figure 1b shows the schematic diagram of the photonic synapse of the membrane system, similar to the human brain synaptic system (Figure 1a). Ge/Si films are used to constitute the synaptic cleft to control the transmission wavelength, and GST film is used to constitute the presynaptic membrane/postsynaptic membrane control weight. A low-energy light λ (tunable wavelength) is fed into the structure, and its transmission level depends on the synaptic weight. A light with a wavelength of 1064 nm (cut-off wavelength) is used to induce the conversion of GST between amorphous and crystalline states, which is used to control synaptic weight. Figure 1c shows a physical picture of photonic synapses prepared on SiO_2_ sheets (2 cm × 2 cm). It is worth noting that the transverse size of the photonic synapse is scalable to the order of nm, which is attributed to the membrane structure. The thickness of the layer is as follows: h_Ge1_ = 90 nm, h_Ge2_ = 200 nm, and h_Si_ = 200 nm.

### 2.2. Methods

First, the transmission matrix method was used to calculate the transmittance of the photonic synapse in the membrane system. Secondly, the finite element software Comsol (version 5.5) was used to analyze the electric field distribution and optical field transmission of the proposed structure. A light field with a standard power of 1 W and a wavelength of 1200–2000 nm was emitted into the photonic synaptic structure. Periodic boundary conditions were used in the model, and the optical constants of the material were obtained from the experimental data, as shown in Table 1.

In the experiments, double-sided polished silica sheets were sonicated sequentially in acetone, absolute ethanol, and deionized water and then blow-dried with N_2_. All films were deposited by a magnetron sputtering instrument (MSP-620, Jinsheng Micro Nano Company, China) in an argon environment with a basic pressure maintained at 1.0 pa for different sputtering powers. Three separate sputtering targets were used for GST, Ge, and Si films (Yipin Chuancheng Technology Company, China): GST (RF target), Ge (DC target), and Si (DC target). The thickness of the film was measured with a 3D non-contact surface profiler (Wyko NT 1100, Veeco, Plainview, NY, USA). A nanosecond laser (YDFLP-C-20, Jpt, Shenzhen, China) was used to induce the phase transition of GST films. The crystallinity was measured by X-ray diffraction (XRD-6100, Shimadzu, Japan), and the complex refractive index was measured by an elliptical polarizer (SE 850 DUV, Sentech, Germany). A spectrophotometer (SP2560 Ni-U, Princeton Instruments, Trenton, NJ, USA) was used to measure the transmission intensity data.

## 3. Results and Discussion

### 3.1. Deposition and Characterization of Thin Film Materials

**Figure 2:**
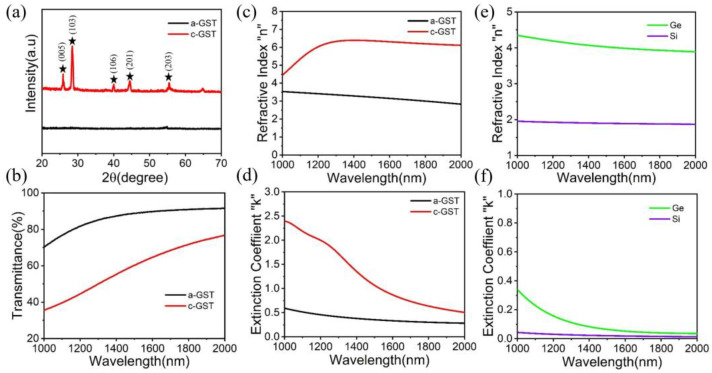
(**a**) XRD data of a-GST and c-GST films, where the star symbol corresponds to the peak position. (**b**) The transmittance of a-GST and c-GST films. (**c**,**d**) The real and imaginary parts of the refractive index of a-GST and c-GST films. (**e**,**f**) The real and imaginary parts of the refractive index of Ge and Si films.

Figure 2a shows the XRD patterns of GST films under annealing. As can be seen from the black line, for the amorphous state (a-GST), there is no obvious characteristic peak. The red line shows that the characteristic peaks (005), (103), (106), (201) and (203) appear after annealing at 200 °C for 30 min, indicating that the GST films are transformed into a crystalline state (c-GST), which is consistent with the literature [27]. Figure 2b–d show that the optical constants of a-GST and c-GST have a great contrast in the wide spectral region. When switching between the two states, the transmittance, refractive index (n value) and extinction ratio (k value) of GST all change dramatically, which is similar to the studies in the literature [28,29]. Figure 2e,f show the real and imaginary parts of the refractive index of Ge and Si. The experimental results show that the refractive index of DC magnetron sputtering Si film is much lower than that of bulk single crystal Si material n value (3.48) [30]. This is attributed to the fact that the crystalline phase structure (non-qualitative) of Si films is different from that of crystalline Si, and the microstructure of Si films is not as dense and compact as that of bulk materials, which leads to the decrease of the refractive index n value. These values are then used to determine the fabricated photonic synaptic devices. 

### 3.2. Layer Structure and Simulation Design of Photonic Synapses

#### 3.2.1. The Layer Structure of Photonic Synapses

**Figure 3:**
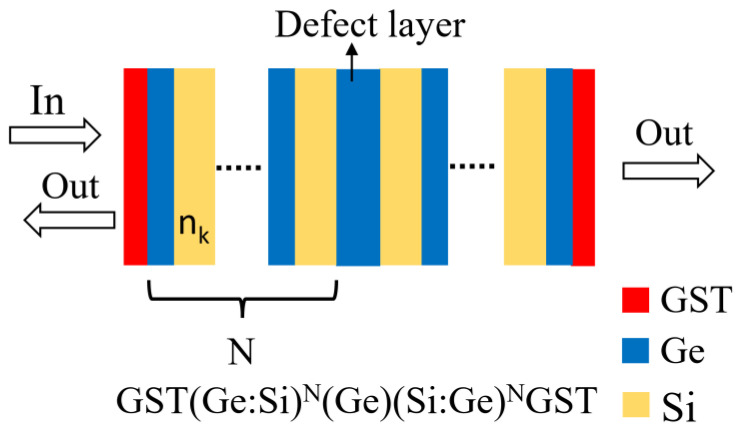
Schematic diagram of the photonic synaptic layer structure.

The schematic diagram of the photonic synaptic layer structure is shown in Figure 3. It is arranged in the manner of GST(Ge:Si)^N^(Ge)(Si:Ge)^N^GST, where N indicates the alternating permutation period of Ge and Si. The photonic synaptic layer structure can be divided into three parts: the phase change layer, periodic layer and defect layer. The phase change layer is composed of GST, which can be induced by laser to produce different phase states. The periodic layer consists of alternating Ge and Si. The defect layer is composed of Ge, and the change in its thickness determines the different transmittance wavelengths. By selecting different photonic synaptic layer structures, the incident light will produce different transmitted light and reflected light, which can realize the light regulation.

#### 3.2.2. Optimal Design of Photonic Synapses

**Figure 4:**
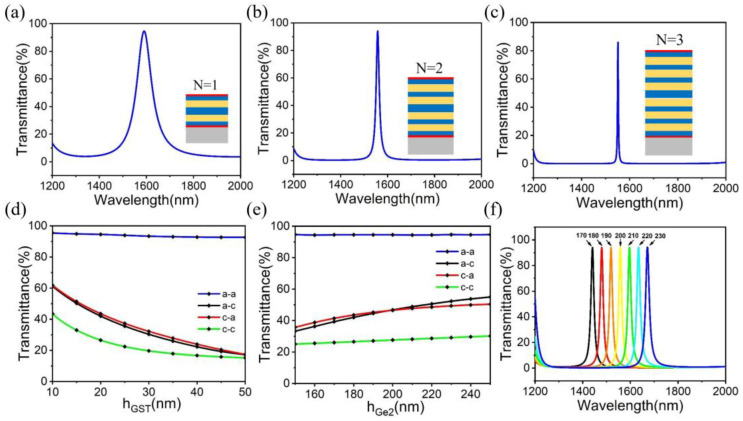
(**a**–**c**) Shows the spectral properties of N = 1, N = 2 and N = 3. (**d**) The effect of GST films on the transmittance of central wavelength. (**e**) Influence of defect layer thickness h_Ge2_ on central wavelength transmittance. Spectrograms at 170 nm, 180 nm, 190 nm, 200 nm, 210 nm, 220 nm and 230 nm are shown in (**f**).

On this basis, the finite element software Comsol is used to study the alternating permutation period (N) of Ge and Si. Figure 4a–c shows the influence of the number of periodic layers (N = 1, N = 2 and N = 3) on the transmission peak. With the increase of N, the half-peak width of the transmission peak gradually narrows, and the center wavelength shifts to the left, while the intensity of the transmission peak decreases slightly, as shown in Table 2. The narrower the half-peak width, the more photonic synapses of different wavelengths can be constructed within a certain band range. However, as the number of layers increases, the preparation of photonic synapses becomes more complex, so the N = 2 structure is finally chosen.

Figure 4d illustrates the effect of the GST film on the transmittance of the central wavelength. When the GST thickness changes from 10 nm to 50 nm, the reduction degree of transmittance is affected by the state of GST. When GST is in the amorphous state (a-a), the transmittance changes to a small extent, but when GST is all transformed into the crystalline state (c-c), the transmittance also decreases rapidly with the increase of GST thickness. In the intermediate state (a-c/c-a), the transmittance changes are the same, which is related to the symmetrical structure of the device. Therefore, different weights can be obtained by selecting the thickness of different GST and controlling the state of GST. Figure 4e shows the influence of the defect layer thickness h_Ge2_ on the transmittance of the central wavelength, and the a-a state spectrograms at the thickness of 170 nm, 180 nm, 190 nm, 200 nm, 210 nm, 220 nm and 230 nm are shown in Figure 4f. Therefore, the photonic synapse at different wavelengths can be obtained by regulating the thickness of different defect layers.

### 3.3. Implementation of a Single Photonic Synapse

**Figure 5:**
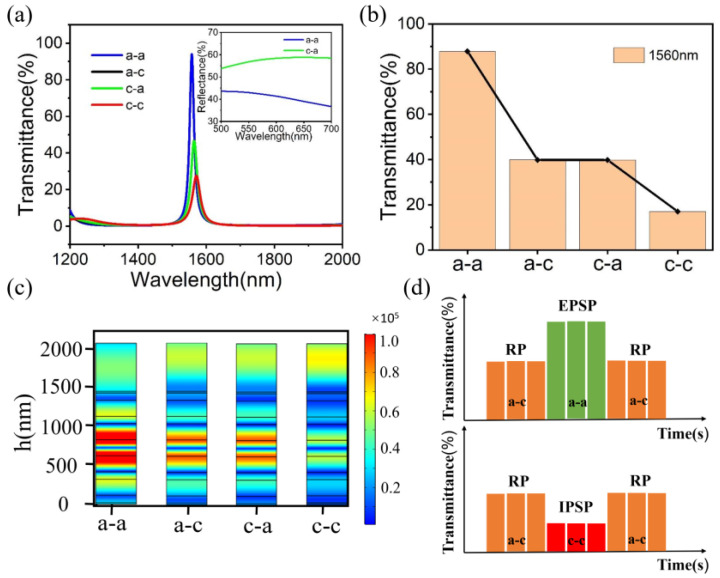
(**a**) Spectral distribution of the photonic synapse. (**b**) The transmittance of photonic synapse at λ = 1560 nm. (**c**) Electric field distribution of photonic synapse. (**d**) IPSP and EPSP of the photonic synapse.

The transmittance spectra of the designed photonic synapse at a-a, a-c, c-a and c-c are shown in Figure 5a. It can be seen that most of the light waves at the photonic synapse are reflected, but a transmission peak is formed at 1560 nm, and the intensity of the peak value changes greatly under a-a, a-c, c-a and c-c. Therefore, 1560 nm is selected as the central wavelength of the photonic synapse. In addition, the difference in reflection spectra between a-a and c-a in the visible range is shown in the upper right corner of Figure 5a, which may provide another possibility for the device to read the signal. In order to simulate the function of the chemical synapse, the photonic synapse is incident with λ = 1560 nm light, as shown in Figure 5b. When both GST films of the photonic synapse are in an amorphous state (a-a), the synaptic structure is highly transmissible and represents a strong connection between the two neurons. The presence of two GST films in opposite states (a-c or c-a) results in an intermediate connection. However, when the two GST films are in the crystalline state (c-c), a weaker connection is caused. This degree of connection represents a different weight. Different GST states lead to different resonances, and the distribution of photonic synaptic electric field intensity under different states is shown in Figure 5c. 

In biological synapses, there are excitatory postsynaptic potentials (EPSP) and inhibitory postsynaptic potentials (IPSP) between pre-synaptic and post-synaptic synapses [31,32]. To achieve this excitatory and inhibitory bipolarity, biological cells need to establish a resting potential (RP) [13]. In this study, the EPSP and IPSP of photonic synapses are obtained by the intensity of light transmittance between pre-synapses and post-synapses. The initial state of the photonic synapse is set to the a-c or c-a state, achieving an RP of an intermediate output (40%). EPSP is obtained by transforming the GST film from the initial state (a-c or c-a state) to a-a state with high transmittance using a 1064 nm pulsed laser. Similarly, IPSP is obtained by transforming the GST film from the initial state to the c-c state with low transmittance. In summary, both EPSP and IPSP can be obtained by changing the state of GST films, as shown in Figure 5d. In this paper, the state of GST is non-volatile, and synapses remain excitatory and inhibitory after the removal of stimulus.

**Figure 6:**
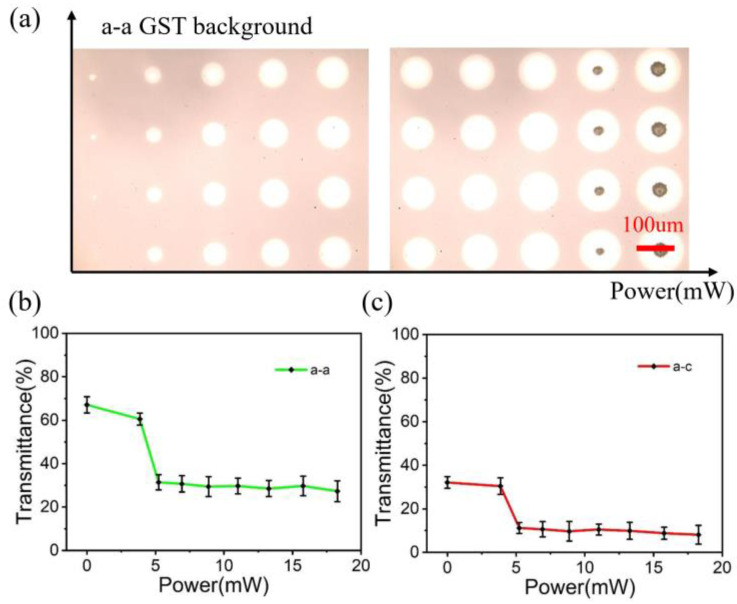
(**a**) Morphology of laser-induced GST phase transition in a-a background. (**b**,**c**) Changes of transmittance with laser power in a-a and a-c background.

In the experimental stage, the initial states of a-c (partially annealed) and a-a (unannealed) samples were prepared by the magnetron sputtering coater and isothermal annealing furnace. Figure 6a shows the phase transition optical morphology of the films in a-a background induced by the nanosecond laser, with optical power ranging from 3.86 mW to 23.39 mW and an action time of 0.5s. With the increase of laser power, the size and brightness of the irradiation point gradually increase. Due to the Gaussian energy distribution of the laser beam, three typical regions can be seen: the amorphous region, the crystalline region and the ablative region. The transmittance of the irradiation point caused by different laser energies is shown in Figure 6b,c. It can be seen that the transmittance remains stable after rapid reduction, indicating that nanosecond laser can achieve the transition from amorphous to the crystalline state, but cannot achieve the induction of the intermediate state. 

### 3.4. Synapse Integration and Neuron Construction

#### 3.4.1. Photonic Synapses Parallel/Series

**Figure 7:**
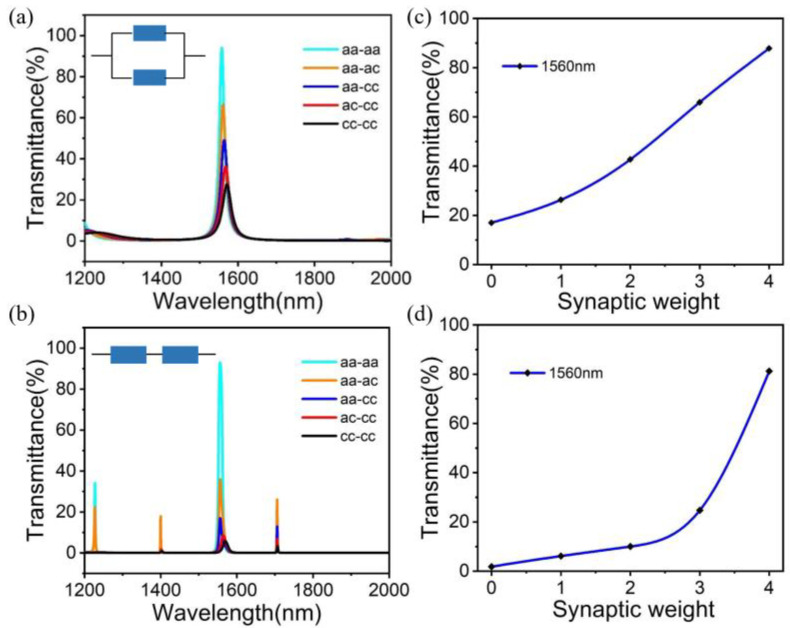
(**a**,**b**) The relationship between GST crystallization rate and transmission peak intensity at photonic synapse series and parallel. (**c**,**d**) The transmittance corresponding to the synaptic weights (0, 1, 2, 3, 4) at the wavelength of 1560 nm.

The construction of complex neural networks requires the integration of hundreds of synapses. In this section, the parallel/series connection of two photonic synapses is demonstrated, which lays a foundation for building more complex neural networks in the future, as shown in Figure 7a,b. We note that by controlling the crystallization rate of GST, different transmission peak intensities can be obtained. At 0% (aa-aa), 25% (aa-ac), 50% (aa-cc), 75% (ac-cc) and 100% (cc-cc) crystallite rates, five different transmission peak strengths are easily obtained. Further, at the wavelength of 1560 nm, more synaptic weights (0, 1, 2, 3, 4) can be easily obtained, as shown in Figure 7c,d. By further increasing the number of photonic synapses, it is possible to obtain more weight and use it to simulate biological systems. It is worth noting that parallel/series corresponds to the principle of single-layer/multi-layer perceptron neural networks. In contrast, the parallel integration method has a similar linear relationship between the weight and the crystallization rate, which lays a foundation for the construction of a single-layer perceptron neural network in this paper.

#### 3.4.2. Construction of a Single Neuron

**Figure 8:**
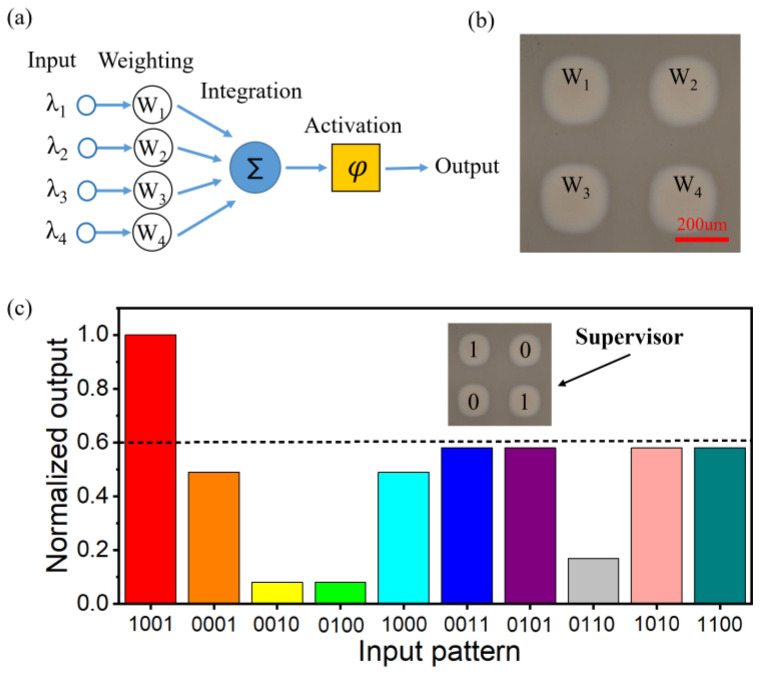
(**a**) Schematic diagram of realizing a single neuron. (**b**) Light microscopic view of a neuron composed of four photonic synapses. (**c**) The normalized output of the neuron is set to “1001” mode.

Figure 8a shows the signal processing of a single neuron in detail [33]. First, the neuronal input signals are weighted by the proposed photonic synapses. Secondly, the detector array is used to detect and sum the signals. Finally, the numerical comparator is used as an activation function for output judgment. It is worth noting that, compared with EPSP/IPSP of photonic synapses, the study of neurons mainly uses EPSP, and the co-implantation of EPSP/IPSP is interested to be investigated in the future. Figure 8b shows the light microscopy of a neuron composed of four photonic synapses. Input signals are input from above the neuron and sent to the detector located below the neuron. 

After implementing an all-optical neuron, the neuron is set to “1001” mode by supervised training (here, synaptic weights are trained by laser pulses). In order to get the appropriate weight value, two methods are applied. A simple network such as the “1001” pattern is constructed by simply swapping weight values. If a complex network is constructed, the predicted output of each training sample should be calculated first. Secondly, the loss function between the predicted value and the output value is constructed. Finally, the gradient descent algorithm is used to calculate the weights of specific applications. After the weight value is obtained, the GST phase transition is induced by a 1064 nm laser to change the photonic synaptic weight, and then verified. The normalized output of neurons is plotted in Figure 8c. It can be seen that when the input signal is 1001 patterns, the neuron is successfully trained to provide the strongest output to the network, and only 1001 output can be obtained by setting the threshold. By increasing the number of photonic synapses and neurons, more complex images can be recognized, as shown below for digital recognition using the same basic method.

### 3.5. Implementation of Single-Layer Neural Synaptic Network

**Figure 9:**
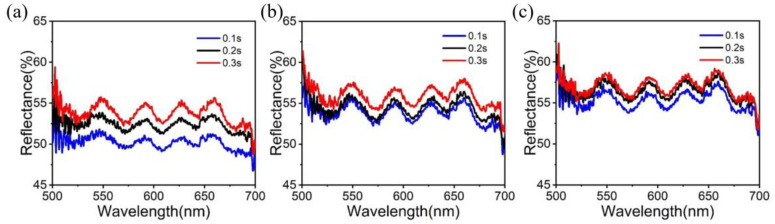
(**a–c**) show the reflectance of the photonic synapses after different laser power (16.18 mW, 22.16 mW and 29.47 mW) and different time (0.1 s, 0.2 s and 0.3 s).

In order to deepen the research of devices, this section first studies the reflection spectrum within the visible range, which is the same as the reflection spectrum in the upper right corner of Figure 5a, hoping to provide another possibility for the device to read the signal. In addition, transmittance and reflectivity are mutually restricted and can be converted into each other regardless of the absorption rate. Figure 9a–c show the reflectance of the photonic synapses under different laser power and at different times. The results show that increasing the laser action time (0.1 s, 0.2 s, 0.3 s) leads to greater reflectivity. In addition, it can be seen from 8a, 8b and 8c that the increase in laser power (16.18 mW, 22.16 mW, 29.47 mW) also leads to the increase in reflectivity. This can simulate the memory function of the human brain, which is affected by the intensity and time of external signal stimulation.

**Figure 10:**
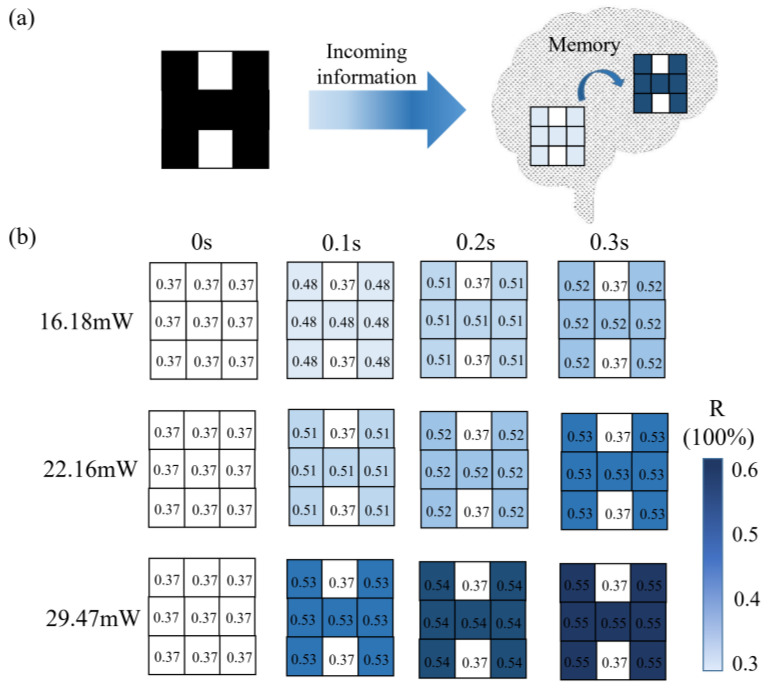
(**a**) Schematic diagram of the cognitive and memory model of the brain. (**b**) The dependence of the reflectance at 680 nm in the letter “H” composed of 3 × 3 pixels on the laser intensity and illumination time.

A typical characteristic of the human brain is its cognitive and memory functions [23], as shown in Figure 10a. Figure 10b shows the simulated human brain memory function of this synaptic network, and the dependence of reflectance on laser light intensity and illumination time is measured at 680 nm. The input image in the letter “H” consists of 3 × 3 pixels, encoded by different light intensities and light illumination times, from which the effects of stimulation light intensities and stimulation times on the synaptic network are compared. In the absence of external stimuli, the reflectance of each pixel is 0.37. Under the action of 16.18 mW and 0.1 s laser, the reflectance of the memory pixel is 0.48. Under 29.47 mW and 0.3 s laser, the reflectance of the memory pixel is 0.55. With the extension of light intensity and time, the degree of memory gradually increased, which corresponded well with the biological memory function.

**Figure 11:**
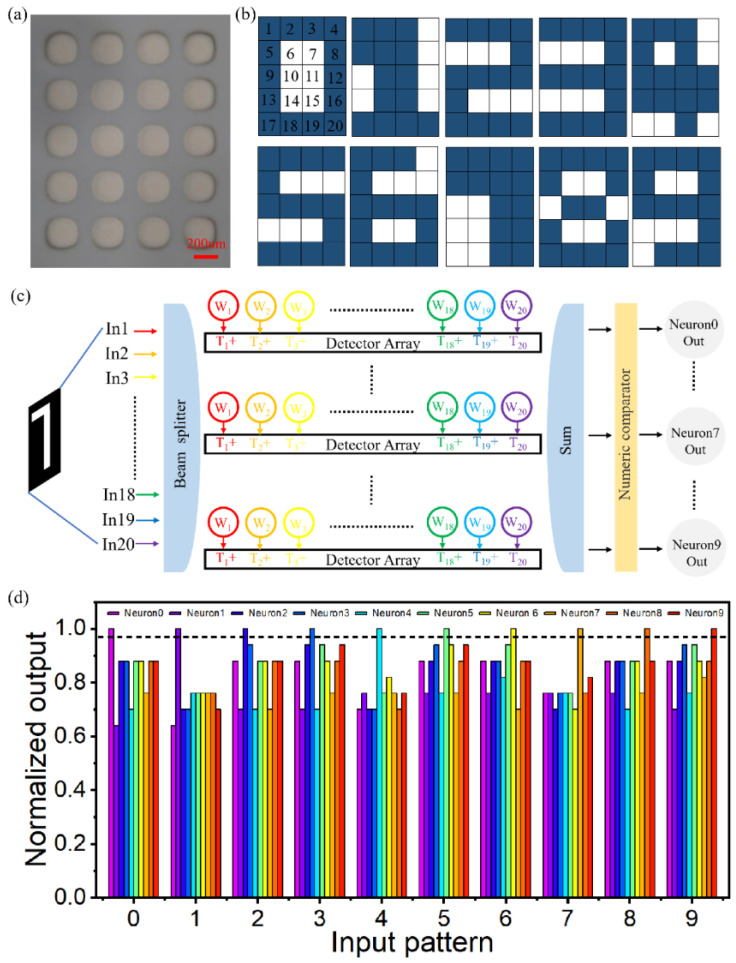
(**a**) Schematic diagram of a neuron composed of 20 synapses. (**b**) The corresponding synaptic weights of the numbers 0, 1, 2, 3, 4, 5, 6, 7, 8 and 9. (**c**) Schematic diagram of single-layer perceptron neural network for numerical recognition. (**d**) The variation of the normalized peak intensity of different input modes with different neurons.

Having demonstrated the ability of individual neurons to remember the letter “H,” we built a single-layer perceptron neural network and tested its ability to recognize numbers. Figure 11a shows a light microscopic diagram of a neuron, consisting of 20 synapses. The single-layer perceptron neural network consists of 10 such neurons and can distinguish the numbers 0, 1, 2, 3, 4, 5, 6, 7, 8 and 9, as shown in Figure 11b. Here, blue represents the highest weight, and the experimental transmittance is about 60%. White represents the lowest weight, and the transmittance is about 10%. A schematic diagram of the working principle of the single-layer perceptron neural network is shown in Figure 11c. First, the output of each neuron is predicted. The GST phase transition is induced by using a 1064 nm laser to change the photonic synaptic weight until it matches the predicted value. Secondly, the optical signals corresponding to the pixel points are fed into the single-layer neural network. Finally, the signal is weighted, summed, and output by different neurons. As expected, neurons are activated only on the corresponding numeric input, as shown in Figure 11d. Neuron 0 fires only when the number “0” is entered, neuron 1 fires only when the number “1” is entered, and so on. 

### 3.6. Discussion

Photonic neural synaptic networks based on optical methods have a broad prospect in computing, which can not only overcome the bottleneck of electronic computing but also have potential advantages such as high speed, low energy consumption and large bandwidth. Previous studies of photonic synapses are mainly based on waveguide-PCMs structures. In our study, we demonstrate a novel membrane-structured photonic synapse and construct a single-layer perceptron neural network. It is worth noting that the transverse size of the photonic synapse is scalable to the order of nm and does not interact with other photonic synapses, which is attributed to the membrane structure. Therefore, the constructed network structure is compact and scalable, and different wavelengths can be obtained by adjusting the thickness of the defect layer of the photonic synapses.

## 4. Conclusions

In conclusion, we propose a novel, but easily integrated, membrane-system structured photonic synapse, where the output signal can be regulated to change the GST films by using laser pulses. The device achieves most synaptic functions, including synaptic weighting, and excitatory and inhibitory bipolar regulation. In addition, the memory function of neurons and the number recognition function of single-layer perceptron neural networks are explored by integrating photonic synapses.

Our work provides a new architecture for integrating photonic synapses, which have advantages such as compact, scalable and tunable spectra, and which can be easily extended and integrated with waveguides or optical fibers, promising large-scale photonic neural synaptic networks.

## Figures and Tables

**Table 1:** Material parameters used in simulations.

	**n**	**k**
a-GST	3.17796	~0
c-GST	6.32731	0.93826
Ge	4.00157	~0
Si	1.89231	~0

All parameters are for 1560 nm.

**Table 2:** The influence period on parameter.

	Half-Peak Width (nm)	Center Wavelength (nm)	Transmission (%)
N = 1	78	1590	94.568
N = 2	16	1558	94.512
N = 3	3.5	1551	91.316

## Data Availability

Not applicable.
